# Antagonism of Macrophage Migration Inhibitory Factory (MIF) after Traumatic Brain Injury Ameliorates Astrocytosis and Peripheral Lymphocyte Activation and Expansion

**DOI:** 10.3390/ijms21207448

**Published:** 2020-10-09

**Authors:** M. Karen Newell-Rogers, Susannah K. Rogers, Richard P. Tobin, Sanjib Mukherjee, Lee A. Shapiro

**Affiliations:** 1 Department of Medical Physiology, Texas A&M University College of Medicine, Bryan, TX 77807, USA; suekrogers3@gmail.com (S.K.R.); mukherjeesanjib@gmail.com (S.M.); 2 Department of Neuroscience and Experimental Therapeutics, Texas A&M University College of Medicine, Bryan, TX 77807, USA; richard.tobin@cuanschutz.edu; 3 B Cell Solutions, Colorado Springs, CO 80907, USA; 4 Department of Surgery, Division of Surgical Oncology, University of Colorado Anschutz Medical Campus, Aurora, CO 80045, USA

**Keywords:** post traumatic epilepsy, immune cells, inflammation, neuroinflammation, adaptive immune response, innate immune response

## Abstract

Traumatic brain injury (TBI) precedes the onset of epilepsy in up to 15–20% of symptomatic epilepsies and up to 5% of all epilepsy. Treatment of acquired epilepsies, including post-traumatic epilepsy (PTE), presents clinical challenges, including frequent resistance to anti-epileptic therapies. Considering that over 1.6 million Americans present with a TBI each year, PTE is an urgent clinical problem. Neuroinflammation is thought to play a major causative role in many of the post-traumatic syndromes, including PTE. Increasing evidence suggests that neuroinflammation facilitates and potentially contributes to seizure induction and propagation. The inflammatory cytokine, macrophage migration inhibitory factor (MIF), is elevated after TBI and higher levels of MIF correlate with worse post-traumatic outcomes. MIF was recently demonstrated to directly alter the firing dynamics of CA1 pyramidal neurons in the hippocampus, a structure critically involved in many types of seizures. We hypothesized that antagonizing MIF after TBI would be anti-inflammatory, anti-neuroinflammatory and neuroprotective. The results show that administering the MIF antagonist ISO1 at 30 min after TBI prevented astrocytosis but was not neuroprotective in the peri-lesion cortex. The results also show that ISO1 inhibited the TBI-induced increase in γδT cells in the gut, and the percent of B cells infiltrating into the brain. The ISO1 treatment also increased this population of B cells in the spleen. These findings are discussed with an eye towards their therapeutic potential for post-traumatic syndromes, including PTE.

## 1. Introduction

Traumatic brain injury (TBI) precedes approximately 15–20% of symptomatic epilepsies and ~5% of all epilepsy cases [1,2]. Post-traumatic epilepsy (PTE) is a frequent consequence of TBI in civilian and military populations, and epidemiological studies have shown that a prior incidence of TBI is a leading cause in the development of delayed symptomatic epilepsy [3,4,5]. Treatment of acquired epilepsies such as PTE presents unique clinical challenges. These patients are often resistant to typical first and second line anti-epileptic drugs, and treatment options are lacking [6,7]. Considering that over 1.6 million Americans present with a TBI each year, PTE is an urgent clinical problem. In addition to the cost of treating the initial injury, which includes missed time from work, school, or service, many people also experience persistent symptoms that can last days, weeks, or even years after the initial injury. The estimated annual cost of TBI, in the United States alone, is in the billions of dollars [8]. Despite ongoing intensive investigations into the mechanisms of injury and the resulting pathology, treatment options are lacking for both initial and chronic post-TBI syndromes. The initial symptoms may include early post-traumatic seizures. Chronic symptoms can include cognitive and affective disorders, as well as the increased susceptibility to developing chronic spontaneous seizures, the hallmark of PTE. 

Neuroinflammation is thought to play a major causative role in many of the post-traumatic syndromes, including PTE. A neuroinflammatory response is rapidly initiated after a TBI, and includes the local brain release of cytokines and chemokines, as well as more widespread activation of inflammatory mediators. A combination of these and other factor can subsequently initiate a peripheral inflammatory response. Collectively, the neuroinflammatory and peripheral inflammatory responses can be neuroprotective in some instances, but can exacerbate injury in other cases [9]. Such exacerbation includes an enhancement of the initial neuronal damage and increase in lesion size. It is unclear to what extent the peripheral immune system contributes to this exacerbation, but some have hypothesized that infiltrating macrophages may be more deleterious than the resident microglial response [9]. Other studies suggest the opposite, that infiltrating macrophages function similarly to the resident microglial cells and may be neuroprotective [10]. Studies also suggest that once a macrophage crosses the blood brain barrier, it assumes a similar function to the resident microglia [10]. Other immune cells may also influence post-traumatic outcomes. 

In human pediatric epilepsy, activated peripheral immune cells were reported in epileptogenic brain areas [11]. Among these, γδ T cells have been recognized as a component of brain infiltrating lymphocytes in pediatric epilepsy and in Rasmussen encephalopathy [11,12]. γδ T cells are a major subset of CD3+ T cells that line the gut epithelium and contribute to intestinal tissue homeostasis, repair and inflammation. Damage to the brain often leads to gastrointestinal dysfunction that is accompanied by chronic inflammation. Furthermore, the gut has been implicated in epileptogenesis [13,14]. Recently, γδ T cells have been shown to contribute directly to gut inflammation as major contributors to inflammatory bowel diseases [15]. Under conditions of stress, γδ T cells are capable of migrating from the epithelium to other sites, including proximal lymph nodes. Our previous work indicated that TBI induces an increase in splenic γδ T cells [16]. However, the repertoire and expansion of gut γδ T cells have not been previously examined after TBI.

In addition to cellular mediators, specific inflammatory cytokines and chemokines have been demonstrated to be pro-epileptogenic [17]. These include IL1β, IL6, TNFα, TGFβ, [6,18,19,20,21,22,23,24,25,26]. Another cytokine that could potentially be involved is macrophage migration inhibitory factor (MIF). MIF is involved in macrophage migration and is elevated following TBI [27]. MIF has been shown to directly alter the structural and firing properties of peripheral [28] and CNS neurons [29], including hippocampal CA1 pyramidal cell dynamics [27]. MIF was originally identified in vitro as a factor that prevents macrophage migration in cell culture assays [30,31], and has also been shown to be a B cell survival factor that promotes proliferation and *migration* of B cells to sites of inflammation [32]. This occurs largely as a part of the innate immune response via MIF-binding to, and signaling through, cell surface CD74 [33]. When MIF binds to CD74, the complex is internalized, and results in downstream innate immune signaling, resulting from activation of the signal peptide peptidase-like 2a (SPPL2a) enzyme [34,35]. The SPPL2a enzyme cleaves CD74 into peptide fragments, including the 42-amino acid peptide, known as the N-terminal fragment (NTF) [34,35]. NTF serves as a transcription factor that stimulates NFκB activity [36] and activation of NF-κB promotes inflammation associated with an innate immune response [37,38].

MIF inhibition has been shown to be neuroprotective in CNS disorders such as stroke [39,40], and MIF interaction with the CD74 receptor has been shown to activate astrocyte responses [41]. MIF-binding to CD74 can be inhibited by administration of the small molecule ISO1 [42,43]. Considering that MIF mediates the migration of immune cells to the site of injury, stimulates astrocyte responses to injury and directly alters neuronal functioning, it could directly contribute to post-traumatic syndromes. Moreover, considering that the extent of MIF elevation after TBI and stroke predict severity and prognosis [44,45], it is possible that inhibiting MIF might be neuroprotective after TBI. Therefore, the following experiments were designed to test the hypothesis that ISO1 administered after a fluid-percussion TBI would decrease neuroinflammation, neurodegeneration, and peripheral immune cell activation and expansion.

## 2. Results

### 2.1. ISO-1 Reduced Astrocyte Activation after fluid percussion injury (FPI), but Had No Significant Effect on Neurodegeneration after FPI

**Figure 1:**
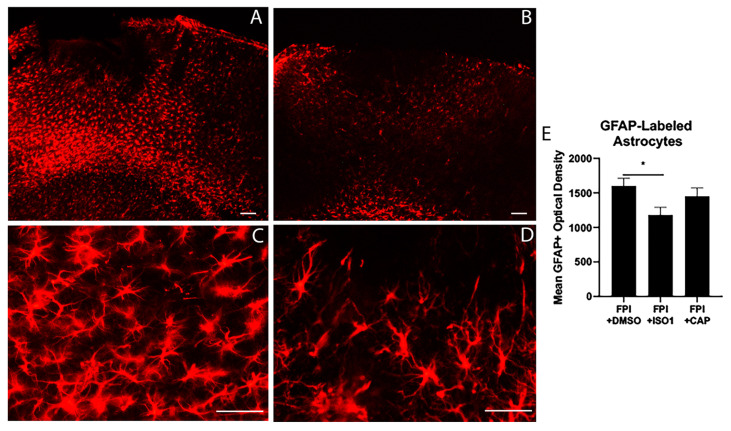
Antagonizing macrophage migration inhibitory factor (MIF) inhibits traumatic brain injury (TBI)-induced astrocytosis. Glial fibrillary acidic protein (GFAP)-labeling in peri-lesion cortex at 3 days after an FPI. In (**A**), GFAP+ astrocytes from an FPI mouse treated with DMSO vehicle at 30 min after FPI. In (**B**), GFAP+ astrocytes from an FPI mouse treated with ISO1 at 30 min after FPI. In (**C**), a higher magnification view or astrocytes from an FPI + dimethyl-sulfoxide (DMSO) mouse to highlight the number and appearance. In (**D**), a higher magnification view of astrocytes from an FPI + ISO1 mouse. Note that FPI causes robust astrocyte activation at 3 days after FPI. Also note that although the ISO1 treatment appears to have reduced the overall GFAP-labeling in this region, some of the astrocytes still appear to be activated. In (**E**), graph of the mean optical density of GFAP-labeling in peri-lesion cortex shows that ISO1 treatment significantly decreased GFAP-labeling, compared to vehicle treated mice (* *p* < 0.05). Scale bars = 250 µm in A and B, and 100 µm in C and D.

**Figure 2:**
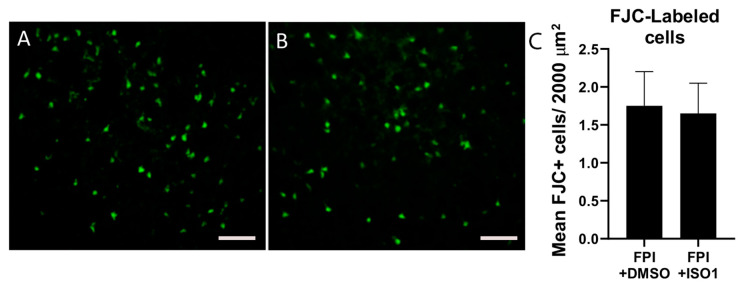
ISO1 has no effect on neurodegeneration at 3 days after FPI. We performed Fluorojade C (FJC) histological staining at 3-days after FPI. In (**A**), FJC-staining in peri-lesion cortex at 3 days after FPI + DMSO. In (**B**), FJC-staining in FPI + ISO mice also reveals robust numbers of degenerating cells. In (**C**), the graph of the means shows that ISO1 had no significant effect on the number of FJC-labeled cells after FPI. This is interesting because we had previously shown that antagonizing the cleaved form of CD74, CLIP, was neuroprotective after FPI. Therefore, is appears as though inhibiting full-length CD74 signaling via MIF antagonism with ISO1 is not neuroprotective, whereas antagonizing a cleaved form of CD74 is neuroprotective. Scale bars = 100 µm.

We assessed the effects of ISO1 (10 mg/kg) on the astrocytic response (Figure 1) and neurodegeneration (Figure 2) in peri-injury cortex, at 3 days after FPI. We found that ISO1 significantly reduced the peak astrocyte response at 3 days post-FPI (Figure 1), whereas it had no significant effect on neurodegeneration after FPI (Figure 2). Therefore, the inhibition of MIF-binding to CD74 results in a decrease in astrocyte activation but has no significant influence on neurodegeneration.

In contrast, when we administered a competitive antagonist peptide (CAP) that antagonizes the proteolytic product of CD74, CLIP, our data showed that administration of CAP at 30 min after FPI significantly reduced the number of degenerating neurons in the peri-lesion cortex [16]. In the present study, we found that administration of CAP at 30 min after FPI had no significant effect on astrocyte activation after FPI (Figure 1). Thus, the impact of inhibiting MIF binding to CD74 using ISO-1 blocked astrocyte activation, while a competitive antagonist to the proteolytic breakdown products of CD74, key components of antigen processing, blocked neurodegeneration. Taken together, the combined results suggest dual contributions of CD74 to astrocyte activation and to antigen processing, respectively: (1) MIF-dependent astrocyte activation which is independent of the proteolytic cleavage of CD74 versus (2) CLIP-dependent contributions to neurodegeneration via the cleavage of CD74 into peptide fragments during antigen processing.

### 2.2. ISO1 Decreased Brain Infiltrating B Cells but Increased Splenic B Cells after FPI

**Figure 3:**
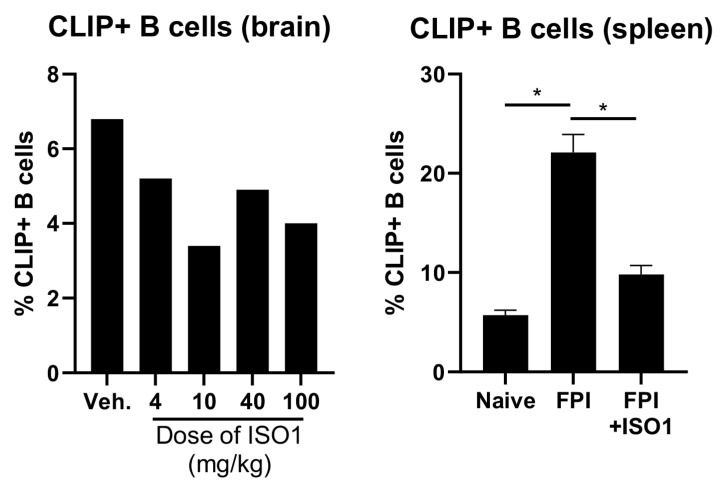
Dose–response curve of ISO1 given at 30 min after FPI, examining immune cell penetration into the brain at 24 h after FPI. We administered ISO1 at 30 min after FPI and performed flow cytometry on leukocytes isolated from the brain to ascertain the optimum dose of ISO1 and to assess what percentage of isolated are CLIP+ B cells. It should be noted that very few cells were isolated from naïve mice, so that was removed from the graph. Cells were stained with anti-CD19 and counter-stained with Anti-MHC class II invariant chain CLIP. The results indicate that a 10 mg/Kg dose of ISO1 administered 30 min after FPI provided the most robust decrease in % of B cells that express CLIP (* *p* < 0.05).

We utilized ISO1, as an inhibitor of inhibit MIF-binding to CD74, to explore the possibility that inhibition of MIF-binding would alter FPI-induced, immune cell infiltration into the brain. We first performed a dose-response curve by injecting different doses (0.2, 2.0, 20 mg/Kg) of ISO1 intraperitoneally 30 min after FPI (Figure 3). At 24 h after FPI, we isolated the white cells using Percoll density gradient centrifugation, and performed flow cytometry on the resulting single cell suspensions to assess what percent of lymphocytes are CLIP+ B cells. It should be noted that less than 0.001 percent of total peripheral B cells enter the brain in untreated, naïve mice. The results show that 10 mg/kg ISO1 at 30 min after FPI provided the most significantly robust decrease in the percent of CLIP+ B cells entering the brain. Reciprocally, we found that ISO1 administration at 30 min after FPI increased the percentage of B cells in the spleen following FPI (Figure 3). Thus, FPI-induced increases in the frequency of B cells that infiltrate the brain, and dose–response curves indicated that this could be reduced by treatment with the MIF inhibitor ISO1. Taken together these data suggest that MIF-antagonism may inhibit migration of peripheral B cells from the spleen to sites of injury, including the brain.

### 2.3. FPI-Induced Increase in the Frequency of γδ T Cells in the Gut Is Inhibited by ISO1

**Figure 4:**
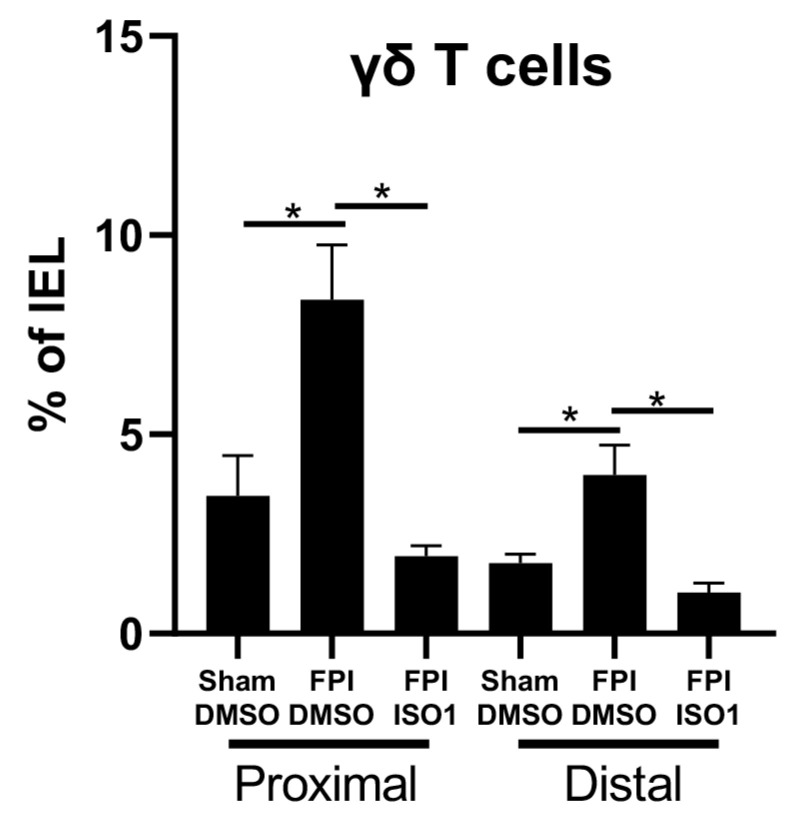
**Effects of ISO1 on the frequency γδ T cells on the gut after FPI.** We administered ISO1 using 10 mg/Kg at 30 min after FPI, and performed flow cytometry on leukocytes isolated from either proximal or distal intestine 24 h following FPI to assess what percentage of isolated leukocytes are γδ T cells. T cells were stained with anti-CD3 antibody and counter-stained with anti-γδ antibody. The percent of T cells that are γδ T cells are significantly increased in the gut after FPI. One way ANOVA * *p* < 0.05. N = 6 per group, error bars = SD.

Damage to the brain can lead to gastrointestinal dysfunction that is accompanied by chronic inflammation. Epithelial γδ T cells represent a major T cell population in the intestine, and likely contribute to intestinal tissue homeostasis and repair. We examined the effects of FPI on the frequency of γδ T cells in the gut and found that FPI significantly increased the percent of γδ T cells in the small and large intestine, and treatment with ISO1 following TBI reversed this effect (Figure 4).

## 3. Discussion

In the present report, we tested the hypothesis that ISO1 will decrease FPI-induced neuroinflammation, neurodegeneration, and expansion of γδ T cells in the gut. We found that blocking the effects of MIF using ISO1 significantly decreased the astrocytic response, but had no influence on neurodegeneration in the peri-injury cortex at 3 days after FPI. We also found that FPI increased γδ T cells in the proximal and distal portion of the intestines and the ISO1 blocked this effect. These results are the first to assess the potential influence of the MIF axis by using ISO1 on the neuroanatomical and gut immune components following FPI, and suggest that MIF may play an important role in post-traumatic inflammation and neuroinflammation.

A major impetus for this study was based on three of our previous observations: first, we reported that MIF can directly alter the firing properties of hippocampal neurons [24]; second, we demonstrated that there is a peripheral expansion of B cells following TBI; and third, we demonstrated that CD74 contributes to neurodegeneration resulting from TBI [16]. In B cells, a small percentage of chondroitin sulfate-modified CD74, approximately 3–5% of the total CD74 in the cell [46], trans-locates to the cell surface independent of its role in antigen presentation and the MHC-II complex. This chondroitin sulfate-modified CD74 acts as the receptor for MIF, which binds to cell surface CD74, signals the recruitment of co-receptor and signaling component CD44 to the complex. MIF binding and the assembly of CD74 with CD44 initiates the downstream inflammatory signaling pathway that results in activation of NF-κB and CD74-dependent B cell survival, proliferation, and migration [47]. It is the initiation of this signaling pathway that contributes to innate immune signaling cascades [47].

Considering these data, an important question was what effect, if any, the MIF antagonist, ISO1, might have on specific cellular components of the immune response to FPI. Our observation that ISO1 selectively inhibited the astrocyte response after FPI suggests that the astrocytic response may be related to innate immune mechanisms that are initiated after an FPI. This result is consistent with recent work linking two distinct morphotypes of astrocytes to neurodegeneration and the dual roles of astrocytosis in neural damage [48,49]. Furthermore, these data indicate that full length CD74, acting in its capacity as a receptor for MIF, contributes to astrocyte activation, but is not required for neurodegeneration. These findings are consistent with previous studies showing that MIF can activate astrocyte responses [41] via its interaction with CD74. The lack of an effect on neurodegeneration suggests that cell death after FPI may involve mechanisms that are not directly related to MIF-stimulated CD74 innate immune signaling but may involve adaptive immune components. Importantly, our finding that ISO-1 reduced GFAP-density may merely be a reduction in proliferation, but not activation of the astrocytes. Previous studies showing that ISO1 inhibits astrocyte proliferation support this notion [50,51]. This might explain why despite ISO1 reducing the overall GFAP-staining, we still observe neurodegeneration in this region. Follow-up studies are needed to fully assess the morphology and activation states of the astrocytes in peri-lesion cortex after FPI.

In addition to immune cell activation, chemical inflammatory cues are also activated following TBI and may contribute to epileptogenesis [9]. Cytokines and chemokines often play multiple and inter-related roles, contributing as growth factors and migratory cues, and as mediators of inflammatory signals. Foresti et al. [52] showed that astrocytes in the hippocampal dentate gyrus up-regulate CCR2, the chemokine receptor for macrophage chemoattractant protein (MCP1). After a chemoconvulsant epileptogenic insult using pilocarpine, these astrocytes were shown to exhibit an altered morphology, such that the orientation of their radial process was directed towards the hilus, rather than towards the granule cell layer [53]. More recently, Robinson et al. [54] showed a similar morphological change to this population of astrocytes following a fluid percussion TBI. In studies of epileptogenesis, these astrocytes were demonstrated to provide an ectopic glial scaffold for the aberrant growth of granule cell basal dendrites into the hilus [53,54,55]. Within the hilus, these aberrant basal dendrites become synaptically targeted by mossy fibers, constituting a pro-epileptogenic, recurrent excitatory circuitry [56,57,58,59,60]. Therefore, the ability of ISO1 to prevent astrocytic alterations after TBI might be a useful target for ameliorating post-traumatic epileptogenesis. The fact that ISO1 has been shown to influence astrocytes in animal models suggest that it can cross the blood brain barrier (BBB). Interestingly, MIF has been shown to increase BBB permeability and vascular leakage, whereas ISO-1 inhibits these effects [40,61]. Still, studies are needed to directly assess the ability of ISO1 to penetrate into the brain.

In our previous study, and here in our current report, we treated a subset of our mice with a competitive antagonist peptide (CAP) of antigen processing and presentation by MHCII. Antigen processing and presentation via MHCII is a central process and the first step in the transition to an adaptive immune response. Using CAP, we previously demonstrated a significant reduction in the increase in neurodegeneration from 1–3 days after FPI. However, treatment with CAP has no influence on the astrocyte response after FPI (Figure 1). These findings support the notion that astrocyte activation after FPI may involve innate immune signaling mechanisms that include MIF signaling through CD74, whereas neurodegeneration after FPI may involve adaptive immune components, involving proteolytic cleavage of CD74 into the fragment CLIP, well established to be a part of antigen processing and presentation. It is important to note that the initial neurodegeneration that is found within 24 h after FPI is likely to involve local, excitotoxic injury [62], but that there is a significant expansion in the lesion size and the number of degenerating neurons from 24 to 72 h after FPI. It is this latter expansion in the neurodegeneration process from 1 to 3 days after FPI that we hypothesize might involve components of an adaptive immune response after FPI.

MIF is known to promote the migration of immune cells. However, we cannot rule out other indirect immune signaling pathways in which MIF might be involved. For example, MIF binding to CD74 is known to cause internalization of CD74. Additionally, MIF binding to CD74 can induce a specific cleavage mechanism of CD74 inside of the cell. These cleavage products of CD74 can induce both innate and adaptive immune signaling components, including the activation of NFkB and involving the activation of lysosomal proteases, respectively. Alternatively, because MIF is known to be a B cell survival factor, it is also possible that inhibiting MIF binding to CD74 might cause CD74+ B cells, and by extension, CLIP+ B cells, to be reduced in numbers or deleted from the B cell repertoire. Therefore, there are several possible explanations for how the MIF:CD74 axis might influence CLIP + B cells in this study. Future studies are needed to fully elucidate these possible mechanisms, as they relate to either innate or acquired immune mechanisms, and especially as they may impact PTE.

TBI is known to affect the brain–gut axis [63]. Γδ T cells represent a key immune component in the gut that regulates intestinal homeostasis and inflammation [64,65], including after CNS injury [66]. We characterized the frequency of γδT cells in the proximal and distal sections of the intestine following FPI (Figure 3). The data revealed that FPI increases the number of γδ T cells both proximal and distal sections of intestine and this can be reversed with ISO1. Gut derived γδ T cells have been shown to be detrimental to stroke outcomes. Therefore, it is possible that despite not improving neurodegeneration, that ISO1 inhibition of MIF after TBI could have other beneficial effects on post-traumatic syndromes.

## 4. Methods

### 4.1. Animals

Eight-week-old male C57BL/6J, were purchased from Jackson Laboratories in Bar Harbor, Maine. The mice were housed at the Baylor Scott and White vivarium facility according to the Institutional Animal Care and Use Committee guidelines (S&W IACUC #2011-059-R).

### 4.2. Fluid Percussion Injury (FPI) Model of TBI

FPI was performed as previously described [67]. Briefly, a 2 mm craniotomy, performed using a stereotaxic device under anesthesia, was performed over the left parietal cortex, keeping dura intact. The female end of a luer-lock syringe was cemented over the craniotomy and attached to the FPI apparatus. A 12–16 ms FPI was delivered at a pressure of ~1.5 atm. Sham mice received identical treatment, with the exception being that no pressure pulse was delivered.

### 4.3. ISO1 Administration

We first performed a dose–response curve (*n* = 5) to determine the optimal dose of ISO1 to use for in vivo studies (Figure 1). Based on these studies, we administered the MIF antagonist ISO1 by administering a single (10 mg/kg) dose of ISO1 intraperitoneally 30 min after FPI. Vehicle groups received the FPI, followed by equal volume injections of saline (the vehicle in which ISO1 is dissolved). The 30-minute post-FPI time point was selected based on our previous study in which we used the CAP peptide to antagonize CLIP-binding to MHCII (Tobin paper). In that foundational study, we selected the 30-minute post-FPI time point because it is clinically relevant and within the timeframe of innate immune responsiveness.

### 4.4. Treatment with Competitive Antagonist Peptide (CAP)

CAP was predicted and synthesized as previously described [68]. Briefly, using computational design, we identified a 9 mer peptide, with a total of 8 amino acid flanking regions, that by, peptide binding analysis software (MHCPred and netMHC), was predicted to have a higher binding constant than the MHC class II invariant peptide (CLIP) for the peptide-binding groove of known MHCII alleles. CAP was synthesized by Elim Biopharmaceuticals. The mice were injected intraperitoneally (i.p.) (1 mg/kg) with CAP. CAP was initially dissolved at 5 mg/mL in dimethyl sulfoxide (DMSO), after which 5 μL of CAP was dissolved in DMSO and then further diluted with 195 μL of sterile saline and injected intra-peritoneally I.P.). Vehicle (DMSO) injected mice received an injection containing 5 μL DMSO dissolved in 195 μL of sterile saline.

### 4.5. Isolation of Brain Infiltrating Leukocytes

Mice were euthanized with isofluorane, followed by full body perfusion through the heart with normal saline. Brains were extracted, homogenized through 100 uM nylon mesh, and resuspended in 30% Percoll. This solution was then layered onto a 70% Percoll solution, centrifuged with no break for 20 min at 500× *g*, and the buffy coat collected and washed using PBS containing 3% fetal calf serum. For all experiments other than the dose–response curve, *n* = 3–6/group.

### 4.6. Isolation of Intestinal Lymphocytes

Mice were euthanized using isofluorane, followed by isolation of intestines. Intestines were thoroughly perfused with normal saline, followed by separation of the intact intestine into proximal (small intestine) and distal (large intestine) components. The intestinal segments were homogenized through 40 μM mesh. Cell suspensions were then layered onto a Percoll gradient and cells between the 1.079 and 1.085 g/mL density were harvested and washed with PBS containing 3% fetal calf serum.

### 4.7. Cell Isolation, Staining, and Flow Cytometry

Single cell suspensions of isolated brain leukocytes, splenocytes, or leukocytes were resuspended, and stained with fluorochrome-conjugated antibodies. Cells were evaluated by surface staining of the with Pacific Blue™ rat anti-mouse CD3e, APC-Cy™7 rat anti-mouse CD19, PE-Cy™7 rat anti-mouse CD8, APC rat anti-mouse MHC Class II (I-A/I-E), PerCP/Cy5.5 rat anti-mouse CD4, and FITC mouse anti-mouse CLIP (15G4) along with LIVE/DEAD^®^ Fixable Aqua Dead Cell Stain. The cells were analyzed on a Becton Dickson FACSCanto II flow cytometer (BD Biosciences Inc., San Jose, CA, USA), consisting of a 3 laser 10 parameter system with FACSDiva software (BD Biosciences Inc., San Jose, CA, USA). The flow data was analyzed using FlowJo^®^ software (FlowJo, LLC, Ashland, OR, USA). For all flow cytometry, samples are coded prior to running through the FACS cell sorter. Once gating strategies have been applied consistently across all groups and data have been collected, the codes are broken for statistical analysis.

### 4.8. Immunohistochemistry and Neuroanatomy

Following FPI, separate groups of mice were perfused with sterile 0.9% saline, followed by 4% paraformaldehyde (PFA) in PBS. Brains were allowed to post-fix in the skull for 24 h, after which they were removed and post-fixed in 4% PFA for 24–48 h, as previously described [69]. Astrocyte activation, and quantification of Fluorojade C (FJC)-labeled cells was assessed. Assessments took place at 3 days post-TBI, a time point when we have previously demonstrated that both, astrocyte activation and neurodegeneration peak in the peri-injury cortex [69]. Astrocytes were identified by staining with CY3-tagged anti-GFAP (Sigma). Stereological quantification of FJC histology in the peri-lesion cortex was performed as previously described [69], and the optical density of GFAP-labeled astrocytes in this region was performed as previously described [24]. For all analyses, slides are coded prior to imaging and codes are not broken until after all data have been collected. Images were systematically captured by a reviewer blind to the condition of the mice, after which a reviewer blind to the condition of the mice performed the analysis on the images.

### 4.9. Statistics

The acquired data from the FlowJo^®^ software were transferred to Microsoft Excel (Redmond, WA, USA) files. After data plotting, statistical significance was analyzed using GraphPad Prism 8 software (La Jolla, CA, USA). Unpaired Student’s t tests were used to compare two groups, and one-way ANOVA with Tukey’s multiple comparisons test was performed for comparison of three or more groups. FJC-labeled cells and GFAP-densitometry were quantified using ANOVA.

## 5. Conclusions

In conclusion, we have demonstrated, for the first time, a strong case implicating MIF/CD74 signaling in the astrocytic response to FPI. We provide circumstantial evidence that CLIP, a cleavage product of CD74 involved in antigen processing and presentation, is involved in processes that lead to neurodegeneration. This suggests a role for adaptive immune components contributing to secondary neurodegeneration that occurs from 24–72 h after FPI. In addition, we provide evidence that FPI causes changes in the frequency of γδT cells in the gut that are MIF/CD74 dependent. Such findings have important implications when considering potential therapeutic options for TBI. Depending on when the treatment is initiated, it is possible that different classes of drugs that can selectively inhibit either the innate and/or the adaptive immune response might maximize the therapeutic potential of such therapies. Indeed, such variance in the immune components of TBI might further explain why clinical trials that incorporate more general anti-inflammatory drugs have thus far failed. Future studies are needed to further define the specific immune components of the immune response, and to test more specific immune inhibitors on specific components of neuroanatomical, neurological dysfunction, and the brain–gut–immune axis following TBI, to prevent post-traumatic epileptogenesis.
